# LMNB1-Related Adult-Onset Autosomal Dominant Leukodystrophy Presenting as Movement Disorder: A Case Report and Review of the Literature

**DOI:** 10.3389/fnins.2019.01030

**Published:** 2019-10-21

**Authors:** Yanyan Zhang, Jie Li, Rong Bai, Jianping Wang, Tao Peng, Lijie Chen, Jingtao Wang, Yanru Liu, Tian Tian, Hong Lu

**Affiliations:** ^1^Department of Neurology, The First Affiliated Hospital of Zhengzhou University, Zhengzhou, China; ^2^Department of Neurology, The Fifth Affiliated Hospital of Zhengzhou University, Zhengzhou, China

**Keywords:** adult-onset autosomal dominant leukodystrophy, *LMNB1* gene, movement disorder, tremor, neurodegenerative disease

## Abstract

Adult-onset autosomal dominant leukodystrophy (ADLD) is a lately described rare form of leukodystrophy with only one family report from China. As the only disease associated with increased lamina B1 encoded by *LMNB1*, ADLDs have different clinical presentations, ranging from autonomic to pyramidal tract and cerebellar ataxia. Here, we report a case of ADLD that presented with positional tremor as the initial symptom. T2-weighted brain MRI showed brain atrophy and diffuse high signal intensity of the cerebral white matter and the brain stem. The precise diagnosis was made by identification of the mutated gene. To the best of our knowledge, this is perhaps the first case report of ADLD presenting as tremor in China.

## Introduction

Leukodystrophies refer to a series of rare genetic, progressive disorders primarily characterized by demyelination or hypomyelination of the central nervous system (CNS), representing approximately the epidemiological frequency of 1/50,000-1/7500 (Kohler et al., 2018). Most leukodystrophies display early onset during infancy and childhood with commonly autosomal recessive or X-linked recessive inheritance. In contrast, only few are currently defined as adulthood leukodystrophies, belonging to dominantly inherited diseases. Adult-onset autosomal dominant leukodystrophy (ADLD) is an autosomal dominant inherited demyelinating disorder, with progressive loss of white matter (WM) in the CNS. Autosomal dominant leukodystrophy is an ultra-rare neurodegenerative disease. Eldridge described it for the first time in Eldridge et al. (1984). Until now, very few number of cases from the United States, Ireland, Sweden, Italy, etc. have been reported (Mezaki et al., 1984; Schuster et al., 1984; Brussino et al., 2009; Dos Santos et al., 2012; Molloy et al., 2012). Evidences to date have pointed that ADLD has been identified as the result of overexpression of lamina B1, and it is the first and only neurological disease related to *LMNB1*, located on chromosome 5q23-31 (Coffeen et al., 2000; Marklund et al., 2006; Padiath et al., 2006; Meijer et al., 2008; Brussino et al., 2010). Under pathological conditions, duplication and deletion upstream of *LMNB1* are two different mechanisms of ADLD to result in the increasing expression and accumulation of laminB1 (Mezaki et al., 1984; Giorgio et al., 2015; Padiath, 2015; Nmezi et al., 2019). LaminB1 is a form of nuclear protein and involved in maintaining nuclear integrity and cellular metabolism processes. Its overexpression can lead to a variety of potent effects, including abnormal development of myelin and alterations in nuclear membrane proteins, nucleus integrity, DNA expression, and localization of nuclear envelope proteins (Ferrera et al., 2014; Bartoletti-Stella et al., 2015; Padiath, 2015, 2016; Giacomini et al., 2016; Liu et al., 2018). Previous studies have further proved that the increased laminB1 appears in different cell types of brain, fibroblasts, or peripheral blood (Brussino et al., 2010), especially oligodendrocytes (Padiath, 2015; Rolyan et al., 2015; Lo Martire et al., 2018). In addition, histology studies revealed loss myelin of cerebral and cerebellar, modest reactive gliosis, preservation of oligodendrocytes, and sparing of inflammation (Coffeen et al., 2000; Melberg et al., 2006).

The onset of ADLD often commences at the age of the fourth or fifth decade with autonomic dysfunction, occurring simultaneously with or followed by pyramidal abnormalities and cerebellar signs (Padiath and Fu, 2010; Lin et al., 2011; Brunetti et al., 2014; Finnsson et al., 2015; Terlizzi et al., 2016; Dai et al., 2017). In several atypical patients, however, autonomic dysfunction could occur after somatic motor dysfunction, or fail to be detected (Brussino et al., 2010; Giorgio et al., 2013; Potic et al., 2013). Some updated reports pointed out that cognitive impairment, auditory or visual abnormalities, cardiovascular and skin noradrenergic failure, and REM sleep behavior disorder (RBD) may be clinical features of ADLD (Guaraldi et al., 2011; Flanagan et al., 2013; Laforce et al., 2013; Sandoval-Rodriguez et al., 2017; Table 1). Compared with the onset of clinical symptoms, MRI findings have been observed about a decade earlier (Finnsson et al., 2015). On conventional MRI, ADLDs are characterized by diffuse and symmetrical lesions in WM and cerebellar peduncles, accompanied by the less-effected periventricular region, optic radiations, and U-fibers (Melberg et al., 2006; Brunetti et al., 2014; Corlobe et al., 2015; Finnsson et al., 2015; Zanigni et al., 2015). Several reports have indicated previously that bilateral abnormal signals in corticospinal tracts, internal capsule, corpus callosum, lemniscus medialis, corticonuclear tracts, and cerebellar peduncles have been observed (Melberg et al., 2006; Finnsson et al., 2013; Potic et al., 2013; Corlobe et al., 2015). Recent studies evidenced decreased brain WM metabolism and pathological sediments of lactate in lateral ventricle CSF in using single-voxel proton-MR Spectroscopy (1H-MRS) (Finnsson et al., 2013, 2019; Zanigni et al., 2015). Furthermore, ADLD is a progressive and fatal disease, and affected people usually survive for 10–20 years after the onset of symptoms (Giorgio et al., 2013; Finnsson et al., 2015). A research demonstrated that the patient with duplication and deletion upstream of *LMNB1* exhibited earlier onset and more severe clinical symptoms (Mezaki et al., 1984).

**TABLE 1:** The clinical manifestations of variant ADLD patients.

**Initial symptoms**	**Family**	**Onset**	**Peculiar symptoms**	**Other clinical symptoms/signs**	**References**
Autonomic dysfunction	Mexico	57	Cognitive impairment	Pyramidal and cerebellar signs	Sandoval-Rodriguez et al., 2017
Motor abnormalities	Serbia	44	–	Pyramidal and cerebellar signs	Potic et al., 2013
	Italy	41–53	Tremor	Pseudobulbar signs	Brussino et al., 2010
Sleep behavior disorder	America	63	–	Pyramidal signs and autonomic dysfunction	Flanagan et al., 2013

The aim of our study was to report a Chinese ADLD patient presenting with postural tremor of the arms as an initial symptom. Brain MRI showed characteristic WM lesion and CNS atrophy.

## Clinical Data

The subject came from a family in Northern China. Written informed consent was obtained from the parents or guardians of the participant for the publication of this case report, and the study was conducted in accordance with the principles of the Declaration of Helsinki and relevant policies in China.

The proband (II-4) had been in good health until she presented mild tremor of hands when she was 58 years old. Firstly, she had the difficulty in using her hands with involuntary movement, soon after gait imbalance, urinary incontinence, and constipation. She was diagnosed as Parkinsonism in another hospital, but symptoms still slowly deteriorated. At the age of 61 years old, she was observed with speech slowly and mild inarticulate speech. Moreover, family history revealed that her father and elder sister had similar gait impairment at least 10 years before death. Her 60-year-old brother had several years’ history of autonomic symptoms and gait disturbance (Table 2), and her nephew was reported to have difficulty walking, but further details were not available.

**TABLE 2:** ADLD age and clinical features at onset.

**Subject (F/M)**	**Age at onset**	**Age at first evaluation**	**Clinical signs at first evaluation**			
			**Autonomic symptoms**	**Cerebellar signs**	**Pyramidal signs**	**Pseudobulbar signs**
II-4 (F)	58	65	Bowel/bladder dysfunction, OH	Action tremor, Ataxia	Positive Babinski signs	Mild inarticulate speech
II-5 (M)	51	60	Bladder dysfunction	Limbs weakness, gait disturbance	Absent	Absent

Comprehensive clinical evaluations were performed when the proband presented on our hospital for diagnosis and treatment. The physical examination on admission revealed that blood pressure was 153/93 and 143/79 mmHg at rest and after standing for 3 min, respectively. On neurological examination, she had postural tremor of all extremities, and increasing frequency and intensity of involuntary movement when keeping arms flat or when in a standing posture. The proband had slightly increased muscle tone and mild spasticity in the lower limbs, but the muscle strength of all extremities was normal. Bilateral-finger-nose and rapid rotation tests, Babinski signs, and Romberg signs were positive. Tilt test result was also positive. Cognitive function measured by Mini-mental state examination (MMSE) was normal.

**FIGURE 1:**
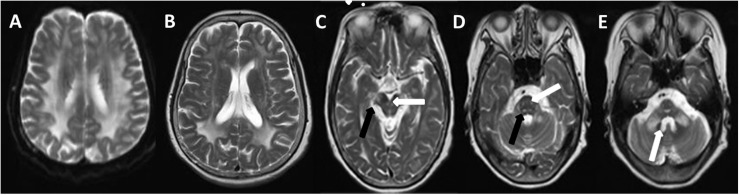
T2W (T2-weighted) changes in the proband. Diffuse hyperintensity of T2W in the white matter of frontals, parietal lobes, basal ganglia, corpus callosum, occipital lobe **(A,B)**, mesencephalon (**C**, arrow), pons, and pons- and cerebellopontine-binding arms (**D**, arrow). The medial lemniscuses, the degustation of the superior cerebellar peduncles (**C**, solid arrow), pyramidal tracts, corticonuclear tracts (**D**, solid arrow), med peduncles, and cerebellar peduncles (**E**, arrow) were also affected.

**FIGURE 2:**
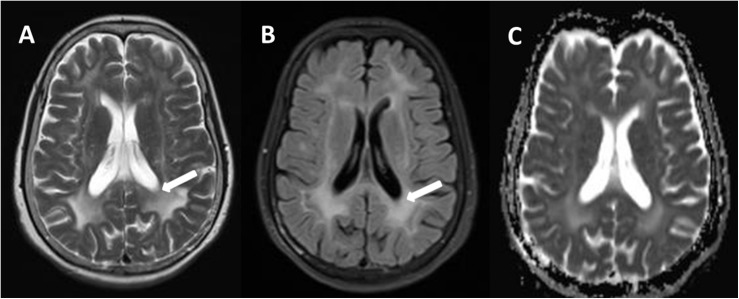
Comparison of a T2W **(A)** and fluid-attenuated inversion-recovery (FLAIR) **(B)**, and apparent diffusion coefficient (ADC) **(C)** in transverse (axial) cross section through hemispheres at lateral ventricles. There are relative preservation of the periventricular region (**A**, arrows) and sparing of the subcortical U fibers on T2W. Compared to T2W, intensity of periventricular rims is suppressed in the most severely affected areas (**B**, arrows). The hyperintense lesion areas in ADC were smaller on T2W **(C)**.

**FIGURE 3:**
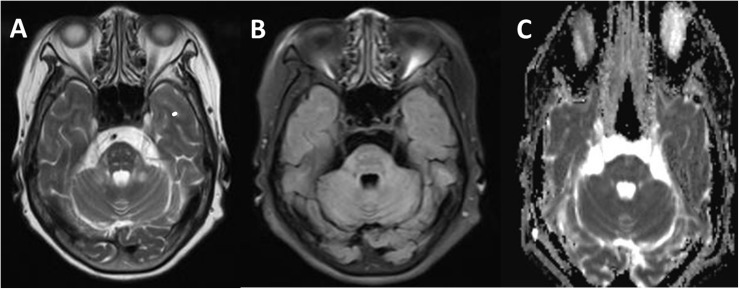
Comparison of a T2W **(A)**, fluid-attenuated inversion-recovery (FLAIR) **(B)**, and apparent diffusion coefficient (ADC) **(C)** in transverse section of the pituitary gland. Signal alterations were extended to pyramidal tracts, corticonuclear tracts, medial lemniscuses, and the degustation of the superior cerebellar peduncles **(A)**. These signals above lesion areas were suppressed on FLAIR and not affected on the ADC map **(C)**.

**FIGURE 4:**
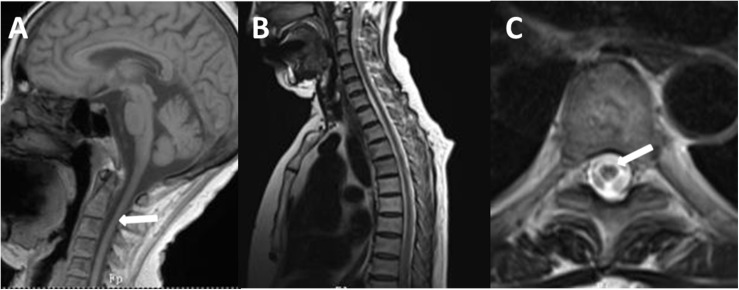
T1-weighted spin-echo image (T1W) **(A)**, T2W **(B)**, and cross-sectional **(C)** MR image of the spinal cord of the proband. T1W on sagittal surface and cross-sectional MRI showed diffuse withering, with accompanying abnormal signal in white matter of thoracic one to four level spinal cord on T2WI.

**FIGURE 5:**
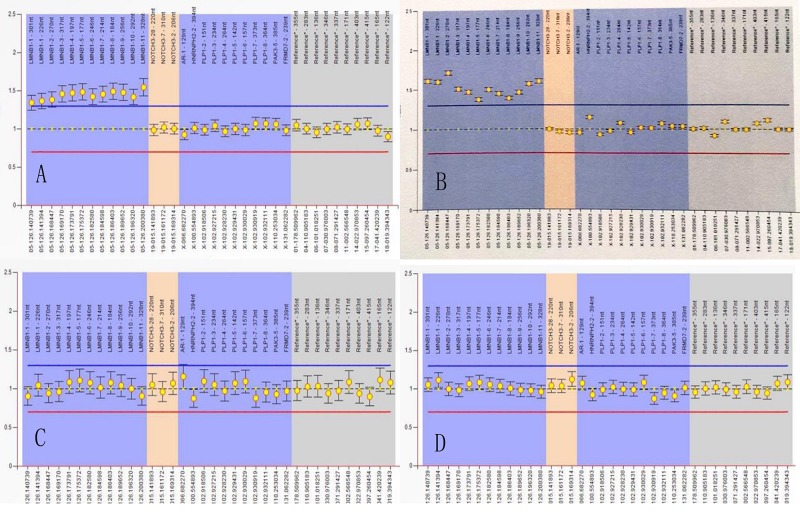
Multiplex ligand-dependent probe amplification (MLPA) experiment. MLPA of II-4 **(A)** and II-5 **(B)** showed genomic duplications of *LMNB1*, extending between nucleotide positions 126.140.739 and 126.200.380, with an estimated size of 59,651 bp **(A,B)**, III-9 **(C)**, and III-11 **(D)** were normal.

**FIGURE 6:**
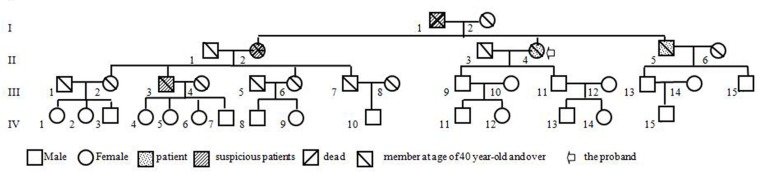
The pedigree of the laminB1-duplicated ADLD family.

The brain MRI scan showed diffuse and symmetrical T2-hyperintense lesions in WM with less affected periventricular rims (Figures 1, 2). Similarly, the cortical spinal tract, cortical nuclear tract, medial thalamus system, and cerebellar foot presented pathological signals on T2-weighted fluid-attenuated inversion-recovery (FLAIR) images (Figure 3). On cross-sectional MRI, the result revealed mild withering of the cerebellum, brain stem, and cerebrum, and diffuse spinal cord atrophy (Figures 4, 5). On the other hand, the genetic tests were in accordance with a diagnosis of ADLD, showing a duplication spanning the entire *LMNB1* gene on chromosome 5q in the proband and her younger brother (Figure 6, II-4, II-5).

## Discussion

Based on clinical feature and neuroimaging data above, the diagnosis of our patient was confirmed by genetic testing. Subsequent to a comprehensive analysis of previous reports, we believe that the female with tremor of limbs as the initial manifestation will be the first Chinese patient.

Tremor is characterized by a rhythmic and involuntary movement of any body part. It commonly has been reported with diseases such as essential tremor, Parkinson disease (PD), multiple sclerosis (MS), and psychogenic and drug-induced tremor (Elias and Shah, 2014). Unlike these diseases, *LMNB1*-related ADLD patients with tremor are extremely rare and ADLDs with tremor as the initial symptom have never been reported previously in China (Dai et al., 2017). There are four studies with patients presenting tremor of limbs or trunk from different countries, including Sweden and Italy (Schuster et al., 1984; Sundblom et al., 2009; Brussino et al., 2010; Terlizzi et al., 2016; Table 3). Analysis result showed that patients from Italy-2 (Schuster et al., 1984), Sweden (Sundblom et al., 2009), and Italy-3 (Terlizzi et al., 2016) were consistent with characteristics of this type of leukodystrophy. They had an onset in about the fifth decade with autonomic dysfunction, characteristic MRI signs, and positive genetic test. By contrast, the Italy-1 family was distinguished from others and defined as ADLD-1-TO because of variant features of the absence of the autonomic dysfunction, relative sparing of cerebellar WM, and the increased laminB1 mRNA without *LMNB1* duplication (Brussino et al., 2010; Giorgio et al., 2015). Although these reports referred to tremor of limbs or trunk, the symptom was thought to be manifestations in the course of disease progression.

**TABLE 3:** Clinical features of individuals affected by ADLD with tremor.

	**Sweden**	**Italy-1**	**Italy-2**	**Italy-3**	**China**
**Clinical findings**					
Age at onset (years)	51	41–53	54	42	58
Initial symptoms	ANS	PS	ANS, PS	ANS, gait impairment	Tremor of limbs
Autonomic dysfunction (constipation, bladder dysfunction, OH)	+	−	+	+	+
Pyramidal signs (hyperreflexia, extensor plantar response, quadriparesis)	+	12/22+	+	+	+
Cerebellar ataxia (Ataxia of limbs)	+	−	+	+	+
Action tremor of limbs	+	9/22+	+	+	+
Intention tremor of limbs	+	N	−	N	
Postural tremor of limbs	+	N	+	−	+
Atactic eye movements	+	N	N	−	−
Pseudobulbar signs	−	8/22+	N	−	+
Sensory loss	−	−	+	−	−
**MRI features**					
White matter lesion in:					
Fronto-parietal lobes	+	+	+	+	+
Internal capsula or pyramidal tracts	+	+	+	+	+
Corpus callosum and fornix	N	±	+	−	+
Upper cerebellar peduncles	+	+	+	+	+
Optic radiations	−	−	N	−	−
Subtle rim of periventricular/subcortical U-fibers	−	−	−	−	−
**CNS atrophy:**					
Fronto-parietal lobes	+	+	−	N	−
Cerebellum	+	±	−	N	−
Medulla oblongata	+	N	N	N	-
Spinal cord	+	N	N	N	+
**Genetic test**					
LMNB1 gene duplication	+	−	+	+	+
LaminBl overexpression	N	+	+	N	N

*ANS, autonomic nervous system; OH, orthostatic hypotension; PS, pyramidal signs; +, sign presence; −, sign absence; N, not be reported.*

Our patient is a peculiar ADLD with the clinical presenting feature being a tremor of limbs. We attribute the movement disorder to ADLD on the basis of the following three pieces of evidence. Firstly, the patient was in good health before first symptoms, excluding the possibility of secondary manifestations of the disease. Secondly, neuroradiological findings display typical clinical signs. T2 high intensities under the cerebral cortex extended down through midbrain, brain stem, to the cerebellum adjacent to the fourth ventricle. Thirdly, and most importantly, gait dysfunction was noted in members over three generations, and five out of nine family members at the age of 40–60 years had similar symptoms, mainly with gait disturbance as the early symptom (Figure 6, I-1, II-1, II-4, II-5, III-3). This study also gets support from gene test, keeping a leading place in the pathogenesis of ADLD.

Unfortunately, present mechanism theories failed to explain the tremor of ADLD in more detail. To the best of our knowledge, the tremor could be associated with neurodegenerative disorders involving the cerebellum and brain structures (Fratkin and Vig, 2012). Moreover, pathological studies suggested that the cerebellum, thalamus, pons, premotor cortical regions, or the basal ganglia may be involved in tremor, especially since the cerebellothalamocortical pathway is essentially involved in all pathologic tremors (Elble, 2013; Muthuraman et al., 2018; Saifee, 2019). Given the above data, we understand that ADLD cases with such heterogeneous presentations had varying degrees of cerebellar lesion. These seem to support the speculation that the demyelination in cerebellothalamocortical network would be responsible for the tremor of ADLD patients. Furthermore, the non-recurrent mutation of *LMNB1* and potential compensatory mechanisms with individual differences may be interpreted as part of the reason why only a few patients present movement disorders in this family (Giorgio et al., 2013).

## Conclusion

To summarize, our study revealed that cerebellar dysfunction presentation may appear as initial onset, and this could be the first case report of ADLD disguising as movement disorder in China. Given the small number of cases and various clinical manifestations, ADLD was often misdiagnosed as other neurodegenerative diseases leading to delay therapy. Neurologists should take comprehensive assessment and take into account the differential diagnosis of patients presenting with movement disorders.

## Data Availability Statement

All datasets generated for this study are included in the manuscript/supplementary files.

## Ethics Statement

The subjects were from a family in Northern China. Written informed consent was obtained from the parents or guardians of the participant for the publication of this case report and the study was conducted in accordance with the principles of the Declaration of Helsinki and relevant policies in China.

## Author Contributions

YZ drafted the manuscript. TT and HL designed the study and TT repeatedly modified the text and details. JL, RB, JPW, TP, LC, and YL participated in the literature review and discussion about article writing and revision. All authors read and approved the final manuscript.

## Conflict of Interest

The authors declare that the research was conducted in the absence of any commercial or financial relationships that could be construed as a potential conflict of interest.
